# Opposite Modulation of Brain Functional Networks Implicated at Low vs. High Demand of Attention and Working Memory

**DOI:** 10.1371/journal.pone.0087078

**Published:** 2014-01-31

**Authors:** Jiansong Xu, Vince D. Calhoun, Godfrey D. Pearlson, Marc N. Potenza

**Affiliations:** 1 Department of Psychiatry, Yale University School of Medicine, New Haven, Connecticut, United States; 2 Child Study Center, Yale University School of Medicine, New Haven, Connecticut, United States; 3 Department of Neurobiology, Yale University School of Medicine, New Haven, Connecticut, United States; 4 The Mind Research Network, Albuquerque, New Mexico, United States; 5 Olin Neuropsychiatry Research Center, Institute of Living, Hartford, Connecticut, United States; 6 Department of ECE, The University of New Mexico, Albuquerque, New Mexico, United States; National Research & Technology Council, Argentina

## Abstract

**Background:**

Functional magnetic resonance imaging (fMRI) studies indicate that the brain organizes its activity into multiple functional networks (FNs) during either resting condition or task-performance. However, the functions of these FNs are not fully understood yet.

**Methodology/Principal Findings:**

To investigate the operation of these FNs, spatial independent component analysis (sICA) was used to extract FNs from fMRI data acquired from healthy participants performing a visual task with two levels of attention and working memory load. The task-related modulations of extracted FNs were assessed. A group of FNs showed increased activity at low-load conditions and reduced activity at high-load conditions. These FNs together involve the left lateral frontoparietal cortex, insula, and ventromedial prefrontal cortex. A second group of FNs showed increased activity at high-load conditions and reduced activity at low-load conditions. These FNs together involve the intraparietal sulcus, frontal eye field, lateral frontoparietal cortex, insula, and dorsal anterior cingulate, bilaterally. Though the two groups of FNs showed opposite task-related modulations, they overlapped extensively at both the lateral and medial frontoparietal cortex and insula. Such an overlap of FNs would not likely be revealed using standard general-linear-model-based analyses.

**Conclusions:**

By assessing task-related modulations, this study differentiated the functional roles of overlapping FNs. Several FNs including the left frontoparietal network are implicated in task conditions of low attentional load, while another set of FNs including the dorsal attentional network is implicated in task conditions involving high attentional demands.

## Introduction

Recent functional magnetic resonance imaging (fMRI) studies demonstrate that the human brain organizes its activities into multiple functional networks (FNs) [1]–[3]. Some FNs show consistent spatial patterns (i.e., involve same key brain regions) across studies using different populations either at resting condition or during cognitive tasks [4]. These FNs include but not limited to the dorsal attention network (DAN), right frontoparietal network (RFPN), left frontoparietal network (LFPN), executive control network (ECN), insula network, temporal network, and anterior and posterior default mode networks (DMNs), though they may have different names in different studies [1]–[3], [5], [6]. Understanding the functions of these FNs will help understand brain functional organization.

Many studies extract FNs from blood-oxygenation-level-dependent (BOLD) signal time series acquired at resting condition, and postulate the functions of different FNs based on their anatomical locations [7]–[10]. However, such postulations may not always be accurate because the function of any given brain region is not fully understood yet. Furthermore, multiple studies, including one from our group, report overlaps of multiple FNs showing different timecourse and task-related modulations [4], [11]–[14]. For example, the DAN, ECN, LFPN, and RFPN all involve the frontoparietal cortex and insula and overlap at both the medial and lateral frontoparietal cortex [4]. They are postulated to exert cognitive control functions [7]–[9]. This raises the question ‘What are the similarities and difference in their cognitive control functions?’.

Several studies have assessed FN modulations during different cognitive tasks. For example, a recent study extracted FNs from fMRI data acquired during a stop-signal task [15] and found that both LFPN and RFPN increased activity at “stop success” trials and decreased activity at “go” trials. However, the two FNs showed opposite modulation at “stop error” trials: activity increased in the RFPN but decreased in the LFPN when participants failed to stop at the stop signal. In another study using a working memory task, the DAN increased activity and the LFPN decreased activity as working memory load increased from a low to a high level [16]. Findings from these two studies indicate that the DAN, LFPN, and RFPN contribute differently to some tasks, but may exert synergistic functions in other contexts. This knowledge of different functions among the DAN, RFPN, and LFPN might not be revealed by analyzing fMRI data acquired at resting condition only.

To further understand the functions of different FNs, this study used spatial independent component analysis (sICA) to extract FNs from an fMRI dataset acquired during a visual target identification task. This dataset has been analyzed using a general-linear-model-based (GLM-based) method in an earlier publication [17]. SICA is one of the most commonly used methods for extracting FNs from BOLD time series [18]. The specific aim of this secondary analysis was to assess the task-related modulations of the timecourses of FNs at low and high task loads separately. Based on findings from our recent study using a similar task [4], we predicted that several FNs including the DAN would show increased activity at high load conditions, while several other FNs including the LFPN would show increased activity at low load conditions.

## Methods

The original study was approved by the Institutional Review Board of University of California Los Angeles (UCLA). The current study employed sICA to extract FNs from an fMRI dataset used in a recent publication, which used SPM2 (Statistical Parametric Mapping, Welcome Department of Cognitive Neurology, London) to assess how different cognitive loads affect distractor-related modulations of BOLD signal in the brain [17]. The sICA approach employed in the current study was similar to the methods used in another recent study [4], which used sICA to extract FNs from an fMRI dataset acquired during a visual target identification task with four levels of task loads, and was focused on the number and spatial extent of FN overlap. The current study was different from [4] by using a different visual target identification task with two levels of task loads and each with two conditions, one with distractors and the other without distractors. The original purpose of this factorial design is to assess task-load effects on distractor related neural correlates (please see [17] for detail). The main aim of the current study was to use sICA to assess FN modulation at low vs. high task load, though FN overlap was also described for the purpose of consistency with findings reported in [4]. Findings in the current study are novel and interesting, and have not been described before. Since the participants, task design, fMRI acquisition, and sICA approach have been described in detail previously [4], [17] they will only be described briefly here.

### Participants

This study included 23 participants (ages 23–41 years, all right-handed, 11 females) recruited from the community of the University of California Los Angeles (UCLA). All participants gave written informed consent to participate in this study, which was approved by the Institutional Review Board of UCLA.

### Task Design

The task used 16 schematic faces as relevant stimuli and 64 scene pictures as background distractors. Faces were composed from different combinations of five facial features (shape of face, eyes, nose, and mouth, and face color). Each facial feature had two different forms (e.g., shapes: round and oval; colors: yellow and blue). The scene pictures were presented as the background of the relevant stimuli. The task used a 2×2 factorial design with two perceptual loads (low, high) and two distractor conditions (with, without). At low load, any face with an oval nose was a target; participants could simply search this feature to identify the target. At high load, targets were defined by face color and shape of eyes, mouth, and face. Participants were required to search for these four features in conjunction to identify the target.

Stimuli for each condition were grouped into blocks with the same 16 faces used in every trial block. These 16 face images, either alone or overlaid on a distractor image, were presented one by one in random sequence within each block. Each image was presented once for 100 ms. The interstimulus interval was 1.1 s, and the duration of each block was 19.2 s. The instruction “Please identify” was presented above an instruction image for 5 s before each trial block. There were four targets randomly positioned in the presentation sequence in each block. During scanning, each subject performed three functional runs using three different task scripts. Within each run, each block condition was repeated four times, and the whole run lasted 387.2 s. In the distractor condition at each load level within each run, each stimulus had a different distractor, but the same 64 pictures were used for both low- and high-load conditions.

### Imaging Data Acquisition

Functional images were acquired using gradient-echo EPI scanning sequence (TR/TE = 1500/30 ms, Flip angle = 70°, 26 slices, 3 mm thick with 1.2 mm skip, 3.1×3.1 mm^2^ in plane pixels) with a Siemens Allegra 3T system. The scanning plane was tilted rostrally from the AC-PC line by 20°. The relatively thin scanning slice and tilted scanning plane were used to reduce susceptibility-related signal loss at the basal forebrain [19]. Each functional run acquired 258 volumes.

### Procedures of Spatial Independent Component Analysis (sICA)

Each BOLD time series was motion-corrected, normalized to the MNI (Montreal Neurological Institute) template, and smoothed with a 5-mm kernel using SPM5 (Statistical Parametric Mapping, Welcome Department of Cognitive Neurology, London). Group ICA (GIFT, http://icatb.sourceforge.net/, version1.3 h) was used to extract spatially independent components (ICs) [20], [21]. The Infomax algorithm was used to extract 23 ICs [22], the optimal number of ICs as estimated by the minimum length description (MLD) criteria [23]. The Infomax algorithm generated a spatial map and a timecourse of the BOLD signal for each IC. This analysis was repeated 50 times using ICASSO for assessing the repeatability of ICs [24]. The stability index of each IC was greater than 0.90 (Fig. S1). Finally, IC timecourses and spatial maps were back-reconstructed for each participant [20], [25], [26].

A systematic procedure was used to diagnose artifacts and FNs. First, the association of each IC’s spatial map with *a priori* probabilistic maps of white matter (WM), cerebrospinal fluid (CSF), and gray matter (GM) in MNI space provided with SPM2 was assessed using GIFT spatial sorting. Six ICs (i.e., 2, 3, 9, 11, 21, and 23) showed high correlations (r^2^>0.05) with CSF or white matter and low correlations (r^2^<0.01) with gray matter indicating that they might be artifacts rather than hemodynamic change. IC18 showed very low correlations (r^2^<0.001) with gray matter and was treated as artifact. Finally, visual inspection revealed that IC19 showed a typical activation pattern of artifact, i.e., a ring of activation around the edge of the brain [27], and was also treated as artifact. Therefore, eight ICs were excluded from further analysis as artifacts (due to head motion, eye movement, ventricular pulsations, etc.).

For defining significant brain regions associated with each IC, we normalized back-reconstructed spatial maps of each IC into z-scores [7], [20]. The z-score of each voxel within a spatial map reflects its contribution to the associated timecourse. The normalized spatial maps of z-scores of each participant were averaged together across the three runs, and the averaged maps of z-scores were entered into second-level random effects analysis (one-sample t test). Therefore, a group level t-map was generated for each IC, and this t-map was used to identify the brain regions involved in the corresponding IC. The significance threshold was set at voxel height p<0.001, False-Discovery-Rate (FDR)-corrected for multiple comparisons of voxel-wise whole-brain analysis (equivalent to t>4.5), and in conjunction with k>5 (where k indicates the voxel cluster extent).

### Assessing Task-related Modulation Over Timecourses

To examine task engagement of each IC, a design matrix for each participant was constructed using SPM5. This design matrix represents the onset of each task block, convolved with a box-car hemodynamic response function. The five-second instruction period before each block was modeled implicitly as task baseline. The temporal sorting tool from GIFT was used to assess engagement of each FN during different task conditions. It uses multiple regression to assess the goodness of fit between the timecourse of each IC and the predicted hemodynamic response function of each task condition. It generates a beta value for each IC and each task condition of each fMRI run. A greater beta value usually indicates a greater engagement of an IC during a specified task condition. For each IC, this regression generated four beta-weight values for each functional run, one for each task condition (i.e., low load without distractors (L), low load with distractors (LD), high load without distractors (H), and high load with distractors (HD)). These beta-weight values represent the correlations between IC timecourses and the canonical hemodynamic response model of task conditions, and index the engagement of ICs during specific task conditions [25]. An increase or decrease in beta-weight values at one task condition relative to another indicates an increase or decrease in task-related activity in the IC.

The beta-weights of each IC for each task condition across three runs of each subject were averaged. The SPSS general linear model (GLM) for repeated measures was used to assess the main effects and interactions of task loads and distractors on the beta-weights of each IC. The significance threshold was set at p<0.05, corrected using Bonferroni-correction for multiple comparisons of 15 ICs (equivalent to p<0.003 before correction). Any ICs surviving this significance threshold were treated as task-related ICs and *post-hoc* one-sample t-tests were performed to assess their beta-weights for each task condition against zero. For these post-hoc analyses, statistical significance was set at p<0.05 without correction for multiple comparisons. A positive beta-weight significantly different from zero indicates a task-related up-modulation of timecourse or increase in activity of the IC during a specific task condition relative to the baseline condition (i.e., task instruction), and a significant negative beta-weight indicates a task-related down-modulation of timecourse or decrease in activity of the IC. Table S2 presents group mean beta-weight values of each IC at each task condition, p values of one-sample t-tests against zero, and p values of load and distractor effects on beta-weights.

### Assessing IC Overlap

For assessing IC overlap, Xjview (http://www.alivelearn.net/xjview8/) was used to convert ICs into binary masks. Only significant voxels surviving the statistical threshold described above (i.e., p<0.001, FDR-corrected, and k>5) were converted into voxels with value of ones in the output mask, while all other voxels were converted into voxels with values of zero. These masks from different ICs were added together. Within the output map, any voxel with a value of two or higher indicated that two or more IC shared this voxel, i.e., overlapped at this voxel.

## Results

### Task Performance

The task performance data have been presented before [17]. In summary, participants showed faster response times (RTs) and lower error rates at low- relative to high-load conditions, indicating that the high-load conditions demanded more attention and working memory.

### Load Effect on ICs

**Figure 1:**
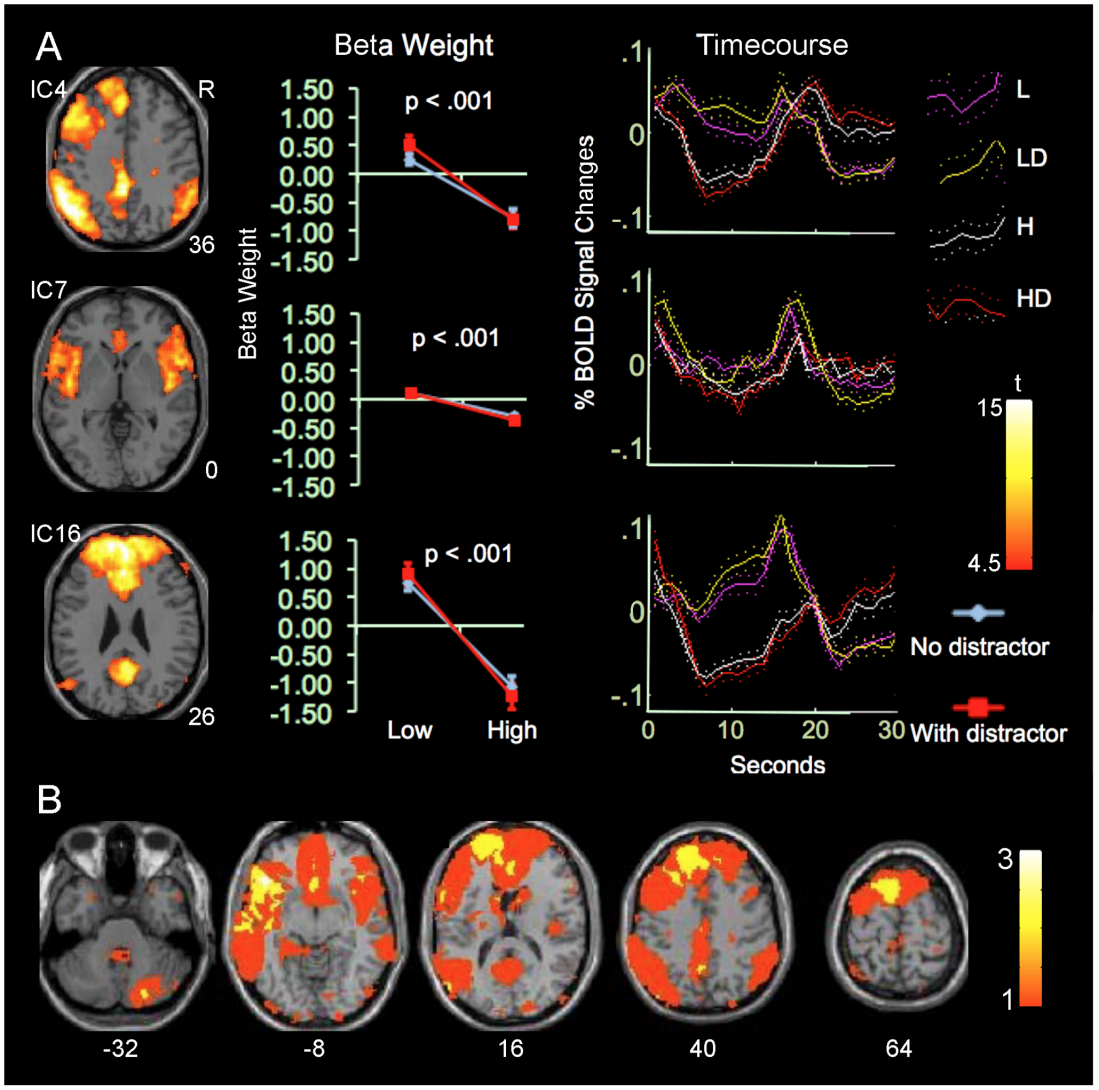
ICs activated at low-load conditions. A. Colors on the Montreal Neurological Institute (MNI) T1 templates show the spatial distributions of positive sub-networks from ICs exhibiting increased activity at low- relative to high-load conditions. Only clusters surviving corrections for voxel-wise whole-brain analyses are shown. The numbers at the bottom right of each brain image indicate Z coordinates in MNI space. The color bar indicates t values. The “Beta-weight” column shows values of beta-weights at low- and high-load conditions. Error bars indicate standard errors (SEs) of the mean. The p value on each panel indicates the statistical significance of the main effect of task load on beta-weight. The “Timecourse” column shows task-load-related modulations in the timecourses of related ICs within 30 s after the onset of task blocks in the four task conditions. For x-axis, 0 represents the onset of task blocks and the block duration is 19.2 s. B. Yellow-red colors on T1 templates indicate brain regions covered by one or more ICs. The color bar indicates the number of overlapping ICs. The number below each brain image indicates the Z coordinates in MNI space. Abbreviations: L: low load without distractors; LD: low load with distractors; H: high load without distractors; HD: high load with distractors; R: right.

Seven of the 15 ICs showed significant main effects of task load. The seven ICs can be divided into two groups based on the direction of load-related modulations. The first group includes ICs 4, 7 and 16 and showed significantly increased beta weights at low- relative to high-load conditions (Fig. 1, Fig. S2 and Table S1). Relative to the control condition, they showed positive beta-weights at low-load conditions, but negative beta-weights at high-load conditions. IC4 involves the left frontoparietal cortex, insula, and precuneus/posterior cingulate (PCC), consistent with the LFPN [4], [6], [15]. IC7 involves the insula and adjacent ventrolateral prefrontal cortex (PFC), perigenual anterior cingulate cortex (ACC), bilaterally, consistent with the insula network [1], [4], [7]. IC16 involves the medial PFC, consistent with the anterior DMN [4], [16], [28], [29].

**Figure 2:**
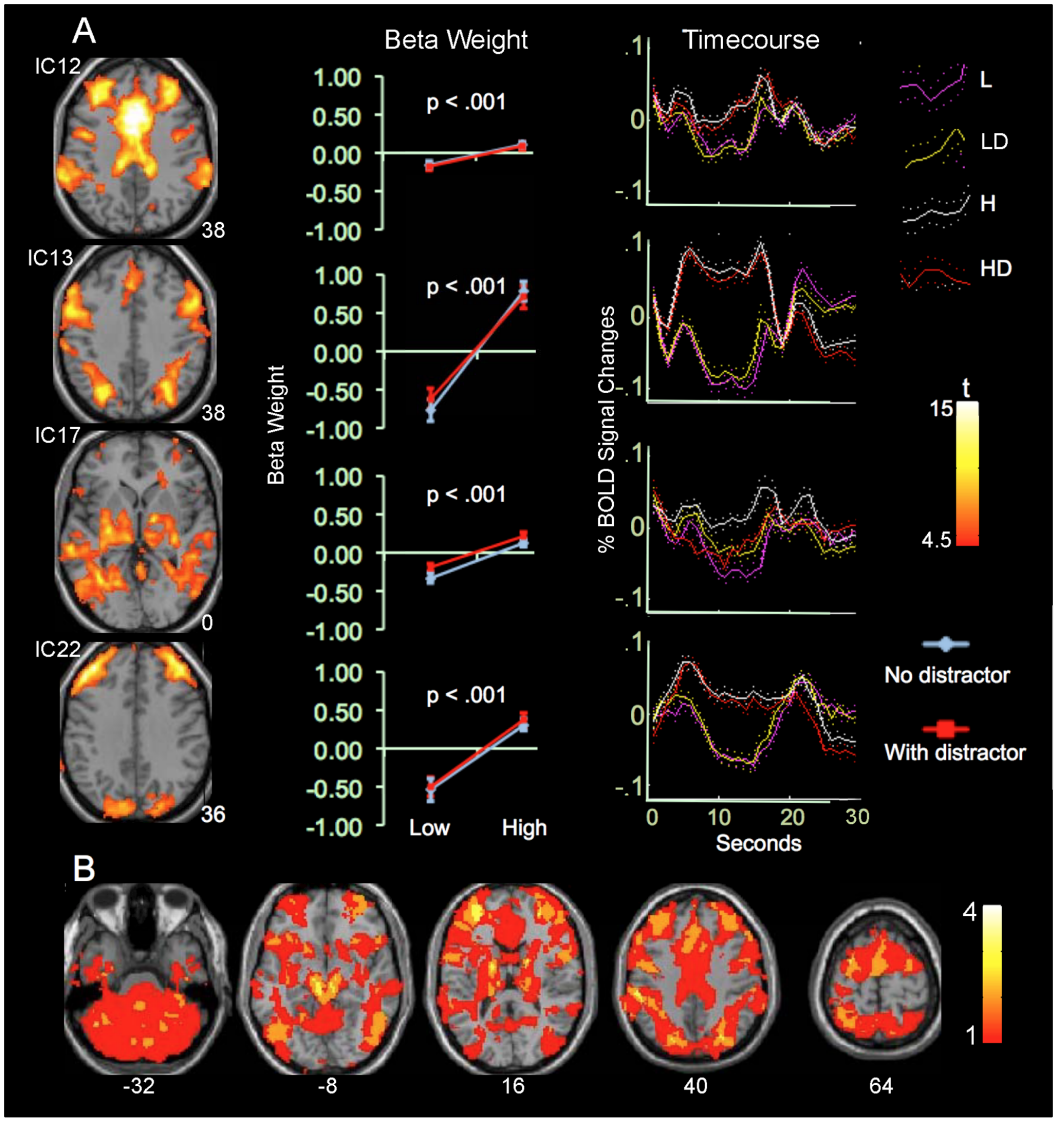
ICs activated at high-load conditions. A. Colors on the Montreal Neurological Institute (MNI) T1 templates show the spatial distribution of positive sub-networks from ICs exhibiting increased activity at high- relative to low-load conditions. B. Yellow-red colors on T1 templates indicate brain regions covered by one or more ICs. Please see fig. 1 legend for additional details.

The second group includes ICs 12, 13, 17, and 22, and showed task-related opposite modulation to the first group. They showed significantly increased beta-weights at high- relative to low-load conditions (Fig. 2, Fig. S2 and Table S1). Relative to the control condition, they showed negative beta-weights at low-load conditions, but positive beta-weights at high-load conditions. IC12 involves the medial and lateral PFC, consistent with the executive control network (ECN) [2], [30]. IC13 involves the frontal eye field (FEF) and intraparietal sulcus (IPS), bilaterally, consistent with the DAN [5], [8], [28]. IC17 involves the cerebellum, thalamus, and striatum, consistent with the cerebellum network [27], [28]. IC22 involves the superior parietal lobule, anterior aspects of the PFC, and caudate heads, bilaterally, consistent with the parietal-frontal network [31], [32].

### IC Overlap

**Figure 3:**
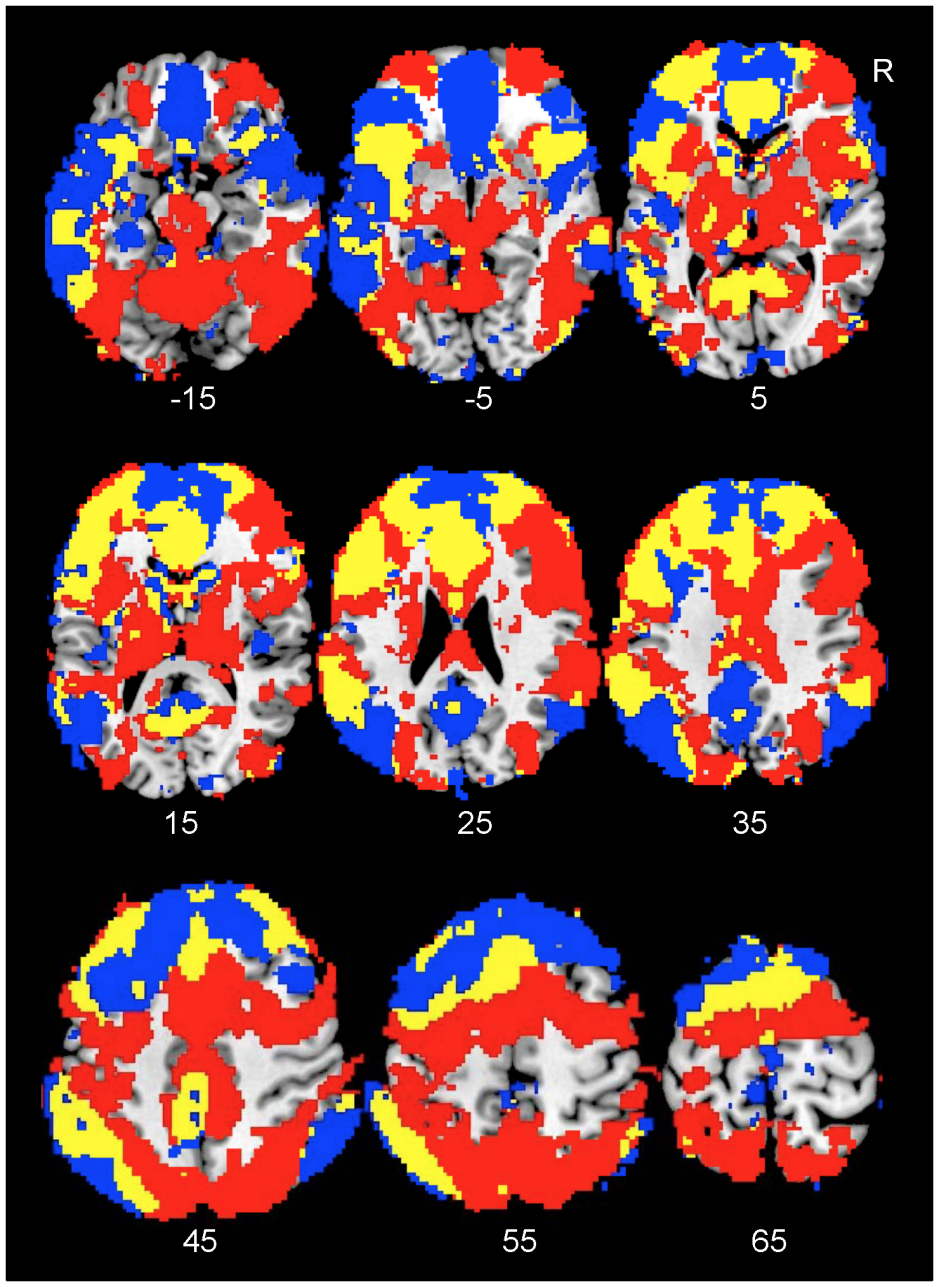
Overlap of ICs activated at low- and high-load conditions. The yellow color on the Montreal Neurological Institute (MNI) T1 templates indicates brain regions covered by overlap of ICs activated at low- and high-load conditions. The blue color indicates brain regions covered by ICs activated at low-load conditions, while the red color indicates brain regions covered by ICs activated at high-load conditions. The number below each brain image indicates the Z coordinates in MNI space. Abbreviation: R: right.

Consistent with our recent findings [4], multiple ICs showed extensive overlap with each other. ICs within the first group overlapped with each other at the medial and lateral PFC, parietal cortex, and ventral temporal cortex. Likewise, ICs within the second group overlapped at these brain regions (Fig. 1 & 2). Furthermore, ICs from the two different groups overlapped extensively at the lateral and medial PFC, parietal and temporal cortex, insula, intraparietal sulcus, thalamus, and striatum, even though they showed task-related opposite modulation (Fig. 3). Relative to the second group, the non-overlapping region of the first group occupied more of the ventral, anterior, and peripheral brain regions including the lateral, anterior, and ventromedial PFC and lateral superior parietal lobule. Relative to the first group, the non-overlapping region of the second group occupied the more dorsal, central, and posterior regions of the brain including the thalamus, basal ganglia, midbrain, posterior PFC, intraparietal sulcus, and insula. Furthermore, the second group covered the cerebellum not covered by the first group. Therefore, the high load conditions activated more brain regions (i.e., the thalamus, basal ganglia, and cerebellum) and more tightly organized brain regions relatively to the low load conditions.

### Distractor Effect on ICs

ICs 1 and 10 showed significant main effects of distractors; i.e., increased beta-weights in the conditions with distractors relative to those without. IC1 involved the medial visual cortex, consistent with the medial visual network (MVN), while IC10 involved the lateral visual cortex, consistent with the lateral visual network (LVN) [1], [27]. ICs 4, 5, 10, and 13 showed distractor-by-load interaction effects on beta-weights; i.e., greater distractor-related increases in beta-weights at low- relative to high-load conditions. However, none of the interaction effects survived correction for multiple comparisons.

### Other ICs

The remaining six ICs (i.e., ICs 5, 6, 8, 14, 15, and 20) did not show significant main effects of task load or distractors (Fig. S2 and Table S1). ICs 5, 6, 8, 15, and 20 were consistent with the sensorimotor network, orbitofrontal network, temporal network, RFPN, and posterior DMN, respectively [1], [3], [5], [6], [28], [33]. To our best knowledge only IC14 was not described in previous publications. The six ICs, except IC14, showed more or less reduced beta-weights during task conditions relative to the control condition, especially during the high-load conditions (Table S1). However, the reductions in beta-weights did not survive correction for multiple comparisons.

## Discussion

Previous studies have demonstrated that some FNs show consistent spatial patterns across different populations [1], [2], [4]. Here, the “consistent spatial pattern” means that the corresponding ICs identified in different studies involve common key brain regions, and do not mean that they are exactly identical [30]. These FNs maintain their general spatial patterns during cognitive tasks with more or less task-related modulations including changes in spatial extents and strengths of internal functional connectivity [27], [30], [34]–[38]. The current study identified 15 ICs. All except one showed spatial patterns consistent with reported ICs, suggesting that our sICA identified appropriately FNs existing in healthy participants.

Relative to our recent study [4], the new data presented in the current study include: 1) explicit description of task-related increases in activity at low task load, but decreases in activity at high task load of several ICs; and 2) detail presentation of task-related decreases in activity at low task load, but increases in activity at high task load of another several ICs. Previous studies have reported “task-negative” and “task-positive” networks, but rarely describe task-related activity increases in “task-negative” networks and task-related activity decreases in “task-positive” networks. The significance of these new findings will be discussed in more details in following discussions. Finally, the new data presented in the current study include the explicit description of different spatial distributions of ICs activated at different task loads.

### ICs Activated at Low-load Conditions

ICs 4 (i.e., LFPN), 7 (i.e., insula network), and 16 (i.e., anterior DMN) demonstrated increased activity at low-load conditions but decreased activity at high-load conditions. This finding was consistent with our prediction that LFPN would show increased activity at low relative to high load conditions. The three ICs together involve the left ventrolateral and ventromedial PFC, TPJ, insula and ACC. Most of these brain regions are associated with the so-called ventral attentional network (VAN), which often shows activity increases during stimulus-triggered bottom-up attentional shifting and activity decreases during tasks with high cognitive loads [8], [39]–[42]. Here, we need to indicate that the definition of VAN is not fully consistent in the literature. Some authors indicate that the VAN involves mainly the right TPJ and ventrolateral PFC [43], others suggest that it involves both hemispheres and includes TPJ, ventrolateral PFC, insula, orbitofrontal cortex, and ACC [41], [44], [45]. The current low-load conditions required participants to identify the targets by searching a feature singleton; i.e., oval nose, among a stream of round noses. Therefore, the low-load conditions may activate the VAN by triggering attention shifting between targets and non-targets. This interpretation of the current finding is consistent with data from several previous studies. First, the LFPF increased activity when stimuli changed from “go” signals to “stop” signals during a go/no-go task and a stop-signal task [15], [33], and decreased activity during a working memory task as working memory load increased from a low to a high level [16]. Second, the insula network increased activity when the standard stimulus changed to an oddball during an auditory oddball task [46]. Finally, the anterior DMN showed stimulus-induced activity during tasks with low cognitive load [47], [48].

The current IC15 is consistent with the RFPN and involves the right TPJ and ventrolateral PFC. These brain regions are main components of the VAN and usually increase activity during stimulus-triggered spatial attentional shifting [8], [43], [49]. In the current study, it showed a tendency of reduced activity during low-load conditions, different from the LFPN showing task-related activity increases, probably due to a lack of spatial attentional shifting during the low-load conditions. Therefore, the LFPN and RFPN exhibit different functional roles during low-load conditions, even though both associated with the VAN. Therefore, the VAN probably involves multiple sub-networks. SICA often separates the DMN into two or three sub-networks [28], [50]. The current study identified two ICs related to the DMN: the anterior and posterior DMNs, respectively. The posterior DMN showed a tendency of reduced activity at low-load conditions, different from the anterior DMN showing activity increases. This finding demonstrates that the DMN is not a homogenous FN.

### ICs Activated at High-load Conditions

ICs 12, 13, 17, and 22 showed activity increases at high- relative to low-load conditions. This finding supported our prediction that DAN (i.e., IC13) would show increased activity at high relative to low load conditions. During the high-load conditions, participants need to keep four facial features in the attentional set and actively search for them in conjunction. Therefore, these task conditions impose a high demand on top-down attentional control, a hypothesized function for the DAN [8], [43], [49]. The current findings indicate that four ICs, not DAN (i.e., IC13) alone, contribute to top-down control. This finding is consistent with the data from several previous studies. For example, the ECN (i.e., IC12) increased activity during an anti-saccadic task [30], the DAN (i.e., IC13) and cerebellum network (i.e., IC17) increased activity as working memory load increased from a low to a high level [16], and the parietal-frontal network (i.e., IC22) increased activity during semantic processing [31]. However, we cannot rule out the possibility that some of the four ICs associate with central processes other than top-down attentional control, such as motor-responses.

Healthy participants often show increased activity in the so-called task-positive networks and decreased activity in the DMN during cognitive tasks, especially those with high cognitive loads [51]–[54]. In the current study, ICs 12, 13, 17, and 20 were task-positive networks because they showed increased activity during high-load conditions. However, they also showed decreased activity during low-load conditions. While task-related deactivation of the “task-positive networks” has not been regularly reported, the current finding is consistent with data from several studies. For example, during a stop-signal task, the LFPN and DAN showed task-related opposite modulations. The DAN was activated at “go” trials but deactivated at “stop” trials while the LFPN was deactivated at “go” trials but activated at “stop” trials [15]. Another study used sICA to extract nine FNs from an fMRI data set. Two of these FNs increased activity during motor sequence learning, while the remaining seven decreased activity. The deactivated FNs involved the dorsolateral PFC and intraparietal sulcus (IPS), typical regions of task-positive networks [55]. Furthermore, the VAN and DAN often show opposite modulations during stimulus-triggered bottom-up attentional shifting [41], [56]. Therefore, the current finding of task-related opposite modulations of ICs 4, 7, and 16 vs. ICs 12, 13, and 17 during low-load conditions is similar to the opposite modulations of the VAN and DAN during stimulus-triggered bottom-up attentional shifting. Such opposite modulations indicate that there may exist intrinsic mechanisms for preventing interruption of central processing by inhibiting competing processes, consistent with the interpretation of opposite modulations of the DMN and task-positive networks [51]–[54].

### IC Overlap

In fMRI, the BOLD signal acquired from each voxel represents a mixture of source signals originated from many different neurons within a voxel and signals from other physiological and non-physiological activities. SICA uses higher-order statistics to extract source signals with independent spatial distributions by separating the signal mixture of each voxel. One of its earliest applications in fMRI demonstrated this unique capability of sICA by separating the BOLD signal mixture from a single voxel into as many as six independent components (ICs) and an average of 3.19 ICs per voxel ([57], fig. 16 legend). Consistent with this finding, several later studies report overlap of FNs generated by sICA, even though each of those FNs shows unique timecourse and/or task-related modulations [15], [27], [30], [38], [46], [58]–[60]. However, this important feature of brain functional organization has been ignored in most published fMRI studies.

More recently three independent groups including us systematically assessed overlap of FNs in four fMRI studies using sICA of two different falvors [4], [11]–[14]. Findings from the four studies together indicate: 1) that multiple FNs overlapped with each other; 2) that overlapping FN showed different, or even opposite task-related modulation; 3) that sICA revealed more extensive brain regions implicated in task performance relative to GLM based analyses; and 4) opposite modulation of overlapping FNs contributed to the negative finding of GLM based analyses at some brain regions. We interpret IC overlap as an indication of the same brain regions supporting multiple cognitive processes simultaneously, consistent with previous interpretations of FN overlap [58]–[60], and predict that the overlap of multiple FNs associated with different cognitive processes will facilitate interactions among them relative to different FNs occupying segregated regions.

The current finding of FN overlap has several important implications. First, previous data on VAN and DAN overlap are not entirely consistent. Several studies report extensive overlap between the VAN and DAN [61], [62], others partial overlap [40], [63], and still others little or no overlap [49], [64]. The current findings are consistent with the data indicating partial overlap. Second, questions exist as to whether FN overlap indicates that the same neural substrates are involved in all overlapping FNs or whether different neural substrates from the same brain regions are involved in different FNs. Animal studies report that different neurons in the same brain region show different timecourses [65], [66], consistent with the notion that different neurons from the same brain region may be involved in different FNs. However, it is possible that the same neurons may be involved in multiple FNs either concurrently or in sequence. Third, the current findings of FN overlap further support previous evidence of there being no sharp border between the DMN and task-positive networks [67]. Finally, the findings indicate that future fMRI studies should identify not only anatomical locations of task-related changes in BOLD signal, but also the FNs implicated in these changes, because BOLD signal from the same locations could be from different FNs with different timecourses and functions.

In addition to their overlap, the non-overlapping regions of the two IC groups show different distributions. Relative to the ICs activated at low load, the ICs activated at high load cover more brain regions including the thalamus, basal ganglia, midbrain, and cerebellum. This finding is consistent with previous evidence that more cerebellar and subcortical regions are recruited as cognitive load increases from a low to a high level [68], [69]. Furthermore, the brain regions activated at the high-load conditions appear to be more tightly organized than those activated at the low-load conditions, suggesting that communications between these regions may be more efficient than communications between regions activated at low load conditions.

FMRI studies often use low load condition as a control and high load condition as a test, and assess neural correlates of brain function under investigation by subtracting task-related activity at low load condition from activity at high load condition [70]–[72]. The implicit assumption of this design is that both low and high load conditions involve the same brain regions and the high load condition activates the brain regions to a greater extent in both amplitude and spatial extent than the low load condition. The current finding indicates that this assumption may not always be correct and investigators should explicitly assess the brain regions activated at different task loads before using this type of study design.

### ICs Activated by Distractors

Two ICs (i.e., 1 and 10) showed increased activity in the conditions with distractors relative to those without distractors. Both ICs involve the visual cortex and therefore this finding is consistent with the data of distractors inducing activity increases in the visual cortex as revealed by GLM-based analysis [17]. Several ICs showed interaction effects of task load and distractor condition on their activity; i.e., distractors induced a greater increase in activity at low- relative to high-load condition. Though this interaction effect did not survive correction for multiple comparisons, this finding is consistent with the data of distractors inducing a greater increase in activity in both the visual and frontoparietal cortex as revealed by GLM-based analysis [17].

### Methodological Consideration

SICA has several limitations. First, the spatial extent of FN overlap is influenced by selected statistical threshold for defining FNs. More strict threshold will lead to less FN overlap relative to less strict threshold. The current study used voxel level p<.001, FDR corrected for the whole brain analysis, and K >5 as threshold. This threshold is very strict relative to the threshold of voxel level p<.01 or.001 without correction, combined with cluster p<.05 corrected for the whole brain analysis commonly used in GLM-based analysis. Second, the spatial pattern of each IC may be different depending on the different number of ICs extracted [73], and the numbers and extent of FN overlap may change for the different numbers of extracted ICs. However, it has been demonstrated that ICs remain accurate for a large range of numbers of ICs [73]. Third, there is no reliable method to accurately identify which IC represents true source signal and which IC represents artifacts generated by ICA. However, many ICs generated by sICA are very consistent in spatial patterns across different studies and populations [2], [27], [30]. Finally, it has been suggested that the constraint of spatially independence of ICs generated by sICA limits its capacity to detect FN overlap [74], [75]. However, this constraint does not mean that different ICs cannot overlap with each other in space. It rather means that the spatial patterns (i.e., spatial distributions in the brain) of different ICs are independent from each other [57], [76].

In conclusion, this study generated important findings by applying sICA to fMRI data acquired during a task with parametric loads of attention and working memory. Two groups of ICs were implicated separately in low vs. high task loads. The first group including ICs 4 (i.e., LFPN), 7 (i.e., insula network), and 16 (i.e., anterior DMN) increased activity at low load conditions and reduced activity at high load conditions. The second group including ICs 12 (i.e., ECN), 13 (i.e., DAN), 17 (i.e., cerebellum network), and 22 (i.e., parietal-frontal network) showed opposite changes of task-related activity relative to the first group, even though the two groups overlapped with each other extensively. Therefore, this secondary analysis of published data revealed that DAN, ECN, and LFPN contributed to cognitive controls at different cognitive demands. The different functional roles of these FNs might not be revealed by studying fMRI data acquired at rest condition alone.

## Supporting Information

Figure S1 **ICASSO results: Stability quality index (Iq) and number of ICA estimates in the estimate-clusters.** The stability index of each component was greater than 0.90.
(DOC)

Figure S2 **Spatial distributions of 15 ICs.** Colors on the Montreal Neurological Institute (MNI) T1 templates demonstrate the spatial distribution of the 15 ICs labeled by their IC numbers. For example, IC1 is the first IC generated by GIFT. The red and blue colors represent positive and negative sub-networks of each IC, respectively. Only clusters surviving corrected voxel height p<0.001 (FDR-corrected for whole-brain analysis) and k>5 are shown. The numbers at top-left of each brain image indicates the Z coordinates in MNI space. The color bar indicates t values. Right side of the brain image is the right side of the brain.
(DOC)

Table S1 **Positive sub-networks of 15 ICs.**
(DOC)

Table S2 **Beta weights of all ICs at each task condition and related p values.**
(DOC)
