# Supplementary material for: Opposite Modulation of Brain Functional Networks Implicated at Low vs. High Demand of Attention and Working Memory
Source: PLoS One. 2014 Jan 31;9(1):e87078. doi: 10.1371/journal.pone.0087078 (PMC3909055; doi:10.1371/journal.pone.0087078)
Supplement: Figure S1 — ICASSO results: Stability quality index (Iq) and number of ICA estimates in the estimate-clusters. The stability index of each component was greater than 0.90. (DOC) [file pone.0087078.s001.doc]

**
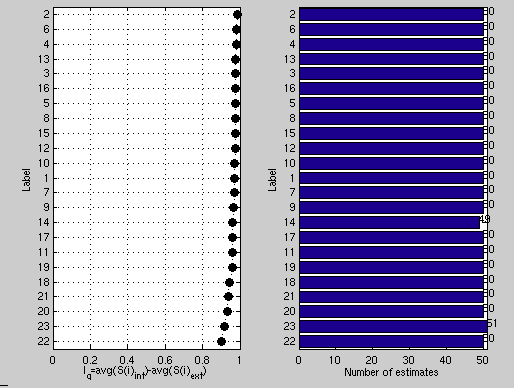
**

Figure S1: ICASSO results: Stability quality index (Iq) and number of ICA estimates in the estimate-clusters. The stability index of each component was greater than 0.90.
