# Supplementary material for: Opposite Modulation of Brain Functional Networks Implicated at Low vs. High Demand of Attention and Working Memory
Source: PLoS One. 2014 Jan 31;9(1):e87078. doi: 10.1371/journal.pone.0087078 (PMC3909055; doi:10.1371/journal.pone.0087078)
Supplement: Figure S2 — Spatial distributions of 15 ICs. Colors on the Montreal Neurological Institute (MNI) T1 templates demonstrate the spatial distribution of the 15 ICs labeled by their IC numbers. For example, IC1 is the first IC generated by GIFT. The red and blue colors represent positive and negative sub-networks of each IC, respectively. Only clusters surviving corrected voxel height p<0.001 (FDR-corrected for whole-brain analysis) and k>5 are shown. The numbers at top-left of each brain image indicates the Z coordinates in MNI space. The color bar indicates t values. Right side of the brain image is the right side of the brain. (DOC) [file pone.0087078.s002.doc]

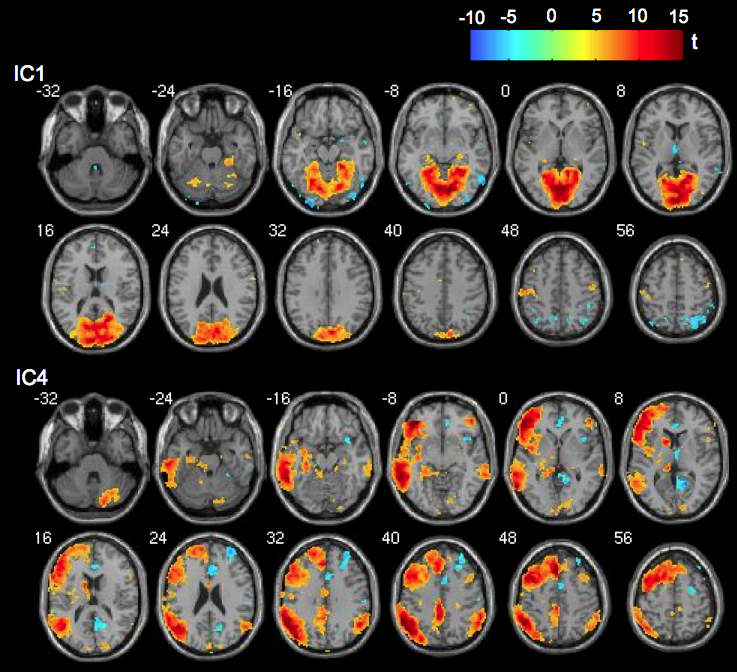

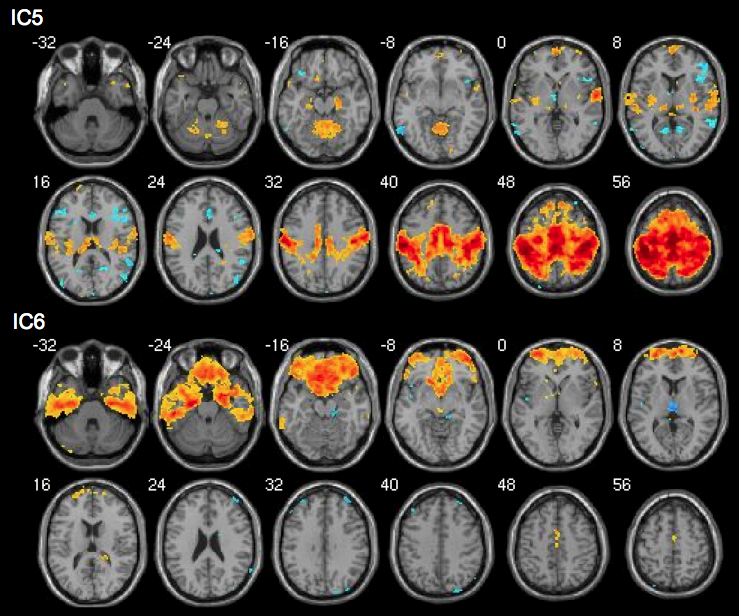


**Fig2 to be continued**

**
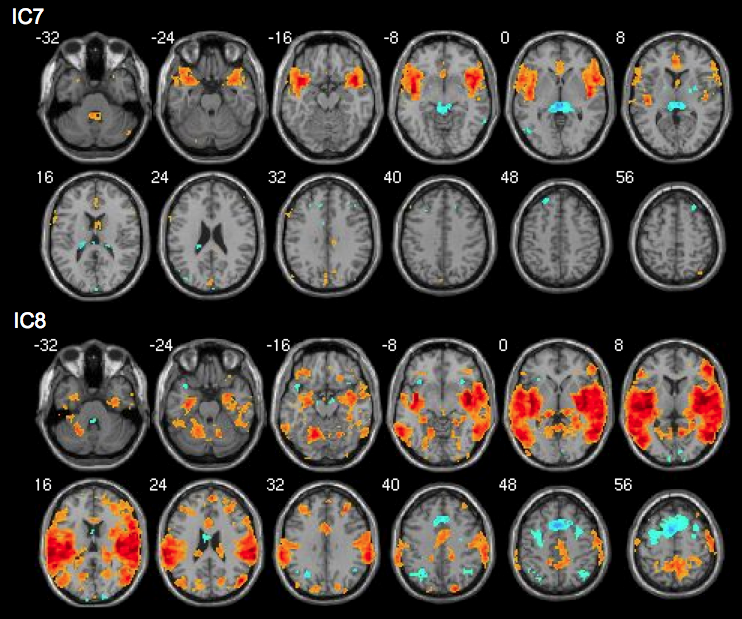

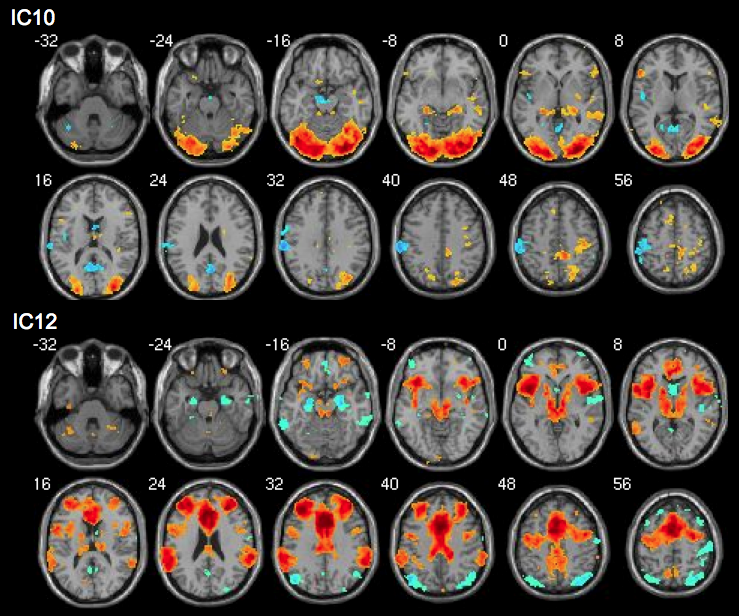
**

**Fig2 to be continued**

**
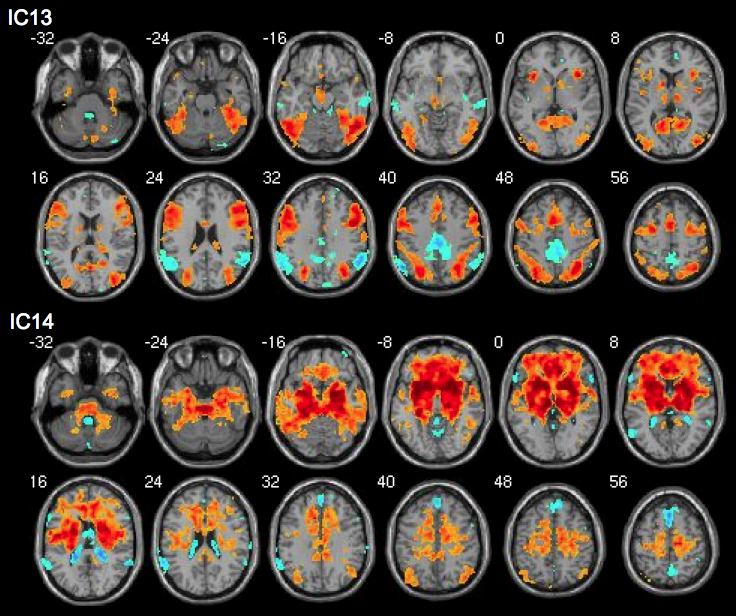

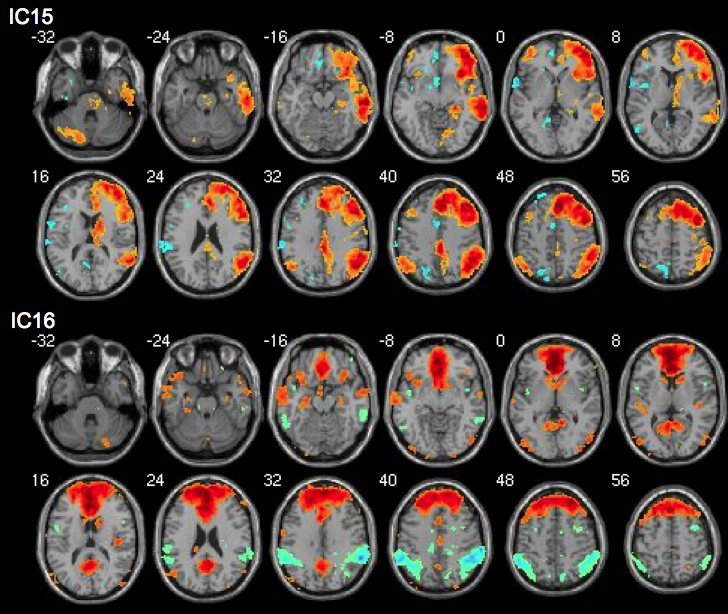
**

**Fig2 to be continued**

**
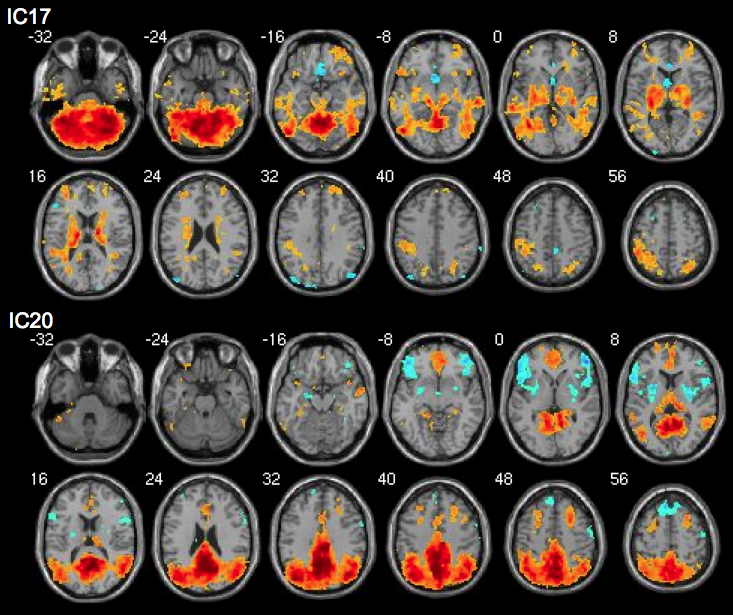

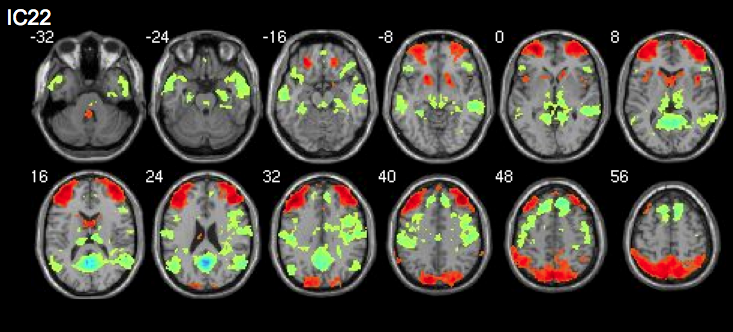
**

Figure S2. Spatial distributions of 15 ICs. Colors on the Montreal Neurological Institute (MNI) T1 templates demonstrate the spatial distribution of the 15 ICs labeled by their IC numbers. For example, IC1 is the first IC generated by GIFT. The red and blue colors represent positive and negative sub-networks of each IC, respectively. Only clusters surviving corrected voxel height p<0.001 (FDR-corrected for whole-brain analysis) and k>5 are shown. The numbers at top-left of each brain image indicates the Z coordinates in MNI space. The color bar indicates t values. Right side of the brain image is the right side of the brain.
