# Supplementary material for: Opposite Modulation of Brain Functional Networks Implicated at Low vs. High Demand of Attention and Working Memory
Source: PLoS One. 2014 Jan 31;9(1):e87078. doi: 10.1371/journal.pone.0087078 (PMC3909055; doi:10.1371/journal.pone.0087078)
Supplement: Table S1 — Positive sub-networks of 15 ICs. (DOC) [file pone.0087078.s003.doc]

Table S1: Positive sub-networks of 15 ICs

|  |  |  |  |  | MNI Coordinates | | |
| --- | --- | --- | --- | --- | --- | --- | --- |
|  | L/R | BA | Size  (voxels) | Z-  Value | X | Y | Z |
| **IC1** | | | | | | | |
| Cuneus, Lingual G., MTG | L/R | 17, 18, 19, 37 | 18346 | >10.0 | 6 | -78 | -6 |
| Pre- & post-central G. | L | 1, 2, 3, 4 | 350 | 5.9 | -60 | -22 | 44 |
| Pre-central G. | R | 4 | 87 | 5.3 | 50 | -10 | 50 |
| **IC4** | | | | | | | |
| SFG, MFG, IFG, ACC,  SMA, Pre-SMA, Insula,  Postcentral G., Inferior &  Superior Parietal Lobule,  Middle Temporal G. | L | 6, 7, 8, 13, 19, 21,  22, 32, 37, 39,  40, 44, 45, 46 | 32207 | >10.0 | -58 | -58 | 38 |
| Precuneus/PCC | L/R | 7, 31 | 2041 | 7.6 | -4 | -46 | 38 |
| Inferior Parietal Lobule | R | 40 | 2019 | 7.1 | 62 | -46 | 40 |
| Caudate | L |  | 394 | 7.7 | -10 | 8 | 12 |
| IFG, MFG | R | 45, 47 | 346 | 6.5 | 48 | 40 | -2 |
| Cerebellum | R |  | 1363 | 6.5 | 18 | -80 | -42 |
| Middle Temporal G. | R | 21 | 754 | 6.2 | 72 | -30 | -12 |
| Cuneus | L/R | 18 | 79 | 5.0 | 6 | -88 | 4 |
| MFG | R | 8 | 297 | 5.1 | 44 | 14 | 50 |
| **IC5** | | | | | | | |
| Pre- & Post-Central G. | L/R | 3, 4, 5, 6, 7 | 42716 | >10.0 | -46 | -30 | 50 |
| Medial SFG and MFG | L/R | 10 | 426 | 5.9 | 0 | 64 | 6 |
| Culmen | L/R |  | 1520 | 5.6 | -16 | -58 | -20 |
| Thalamus | L |  | 298 | 5.6 | -12 | -24 | 6 |
| Hippocampus | R |  | 112 | 5.1 | 22 | -14 | -16 |
| Thalamus | R |  | 181 | 4.8 | 14 | -22 | 4 |
|  |  |  |  |  |  |  |  |
| **IC6** | | | | | | | |
| Hippocampus, Medial SFG,  IFG, OFC | L/R | 10, 11, 25,  34, 47 | 20033 | 6.8 | -22 | -10 | -22 |
| Cerebellum | L/R |  | 753 | 5.5 | 40 | -80 | -46 |
| Mid-Cingulate G. | L/R | 6, 24 | 115 | 5.1 | 0 | 2 | 48 |
| **IC7** | | | | | | | |
| Insula | L | 13 | 4789 | 7.3 | -46 | -4 | -8 |
| Insula | R | 13 | 4023 | 7.2 | 44 | 16 | -16 |
| Peri-genual ACC | L/R | 24, 32 | 642 | 5.6 | -2 | 40 | 8 |
| **IC8** | | | | | | | |
| MFG, IFG,  Motor Cortex, Insula, Inferior  Parietal Lobule, Superior &  Middle & Inferior Temporal G.,  Lingual G., Thalamus, Putamen | L/R | 4, 13, 18, 22,  37, 39, 40, 41,  42, 44, 45, 46, | 53531 | >10.0 | 40 | -14 | 12 |
| MFG | L | 11 | 292 | 5.5 | -40 | 34 | -14 |
| MFG | R | 11 | 94 | 4.9 | 24 | 26 | 42-18 |
| **IC10** | | | | | | | |
| Middle Occipital Gyrus, Lingual  Gyrus, | L/R | 17, 18, 19 | 17996 | >10 | 30 | -88 | -16 |
| Thalamus | R |  | 424 | 6.2 | 24 | -28 | -4 |
| Thalamus | L |  | 378 | 6.0 | -24 | -30 | -2 |
| IFG | L | 45, 47 | 183 | 5.8 | -54 | 26 | 6 |
| STG | R | 22 | 753 | 5.4 | 50 | -14 | -2 |
| Medial SFG | L | 8 | 170 | 5.2 | -4 | 24 | 52 |
| IFG | R | 46 | 205 | 5.0 | 60 | 32 | 2 |
| **IC12** | | | | | | | |
| mSFG, ACC | L/R | 24, 32 | 36583 | >10.0 | -4 | 18 | 30 |
| Inferior Parietal Lobule | L | 40 | 2903 | 7.2 | -58 | -40 | 22 |
| Inferior Parietal Lobule | R | 40 | 1831 | 7.1 | 64 | -48 | 34 |
| SFG | R | 11 | 219 | 5.1 | 36 | 52 | -16 |
| SFG | L | 11 | 155 | 5.0 | -28 | 46 | -16 |
| **IC13** | | | | | | | |
| ITG, IFG, MFG,  Superior Parietal Lobule,  Inferior Parietal Lobule | R | 7, 9, 19, 20,  37, 40 | 13185 | 7.8 | 54 | -60 | -18 |
| ITG, IFG, MFG,  Superior Parietal Lobule,  Inferior Parietal Lobule | L | 7, 9, 19, 20,  37, 40 | 16813 | 7.4 | -50 | -58 | -20 |
| ACC, SMA, Pre-SMA | L/R | 6, 8, 24, 32, | 1592 | 7.3 | -2 | 8 | 48 |
| Putamen, Caudate Head | L |  | 318 | 6.2 | -12 | 8 | 6 |
| Insula | R | 13 | 134 | 6.0 | 44 | -4 | 10 |
| Thalamus | R |  | 375 | 5.8 | 8 | -12 | 6 |
| Thalamus | L |  | 153 | 5.8 | -8 | -16 | 4 |
| Midbrain | L/R |  | 428 | 5.6 | -6 | -20 | -8 |
| OFC | L | 11 | 187 | 4.9 | -1 | 20 | -18 |
| **IC14** | | | | | | | |
| Basal Ganglia, Insula,  OFC, thalamus, Midbrain | L/R | 10, 11, 13 | 60414 | >10.0 | 18 | 4 | -12 |
| Inferior Parietal Lobule | R | 40 | 615 | 5.6 | 48 | -60 | 34 |
| Inferior Parietal Lobule | L | 40 | 900 | 5.6 | -34 | -76 | 36 |
| **IC15** | | | | | | | |
| mSFG, ACC, IFG, MFG | R | 6, 10, 24,  32, 45, 46 | 32668 | >10.0 | 8 | 36 | 48 |
| Precuneus/PCC | L/R | 31 | 1639 | 7.3 | 0 | -32 | 34 |
| Thalamus | R |  | 925 | 6.6 | 6 | -12 | 4 |
| Inferior Parietal Lobule | L | 40 | 1190 | 6.3 | -48 | -62 | 42 |
| MFG | L | 10 | 495 | 5.8 | -38 | 56 | 2 |
| MFG | L | 9 | 374 | 5.8 | -42 | 22 | 40 |
| Insula | L | 13 | 101 | 4.8 | -30 | 22 | -10 |
| **IC16** | | | | | | | |
| SFG, MFG, SMA, Pre-SMA,  Peri-genual ACC,  Caudate | L/R | 8, 9, 10,  24, 32, 33 | 23646 | >10.0 | -2 | 36 | 6 |
| PCC/Precuneus | L/R | 23, 31 | 2734 | 6.9 | 10 | -50 | 2 |
| Insula | L | 13 | 749 | 6.3 | -32 | 10 | -12 |
| MTG. | L | 21 | 1173 | 6.2 | -58 | -12 | -12 |
| IFG | R | 47 | 340 | 5.3 | 32 | 16 | -12 |
| Hippocampus | L | 36 | 248 | 6.0 | -28 | -14 | -18 |
| Insula | R | 13 | 168 | 5.9 | 42 | -22 | 12 |
| Hippocampus | R | 36 | 118 | 5.9 | 28 | -12 | -18 |
| **IC17** | | | | | | | |
| Cerebellum | L/R |  | 45214 | >10 | -4 | -56 | -22 |
| Medial SFG | L/R | 6 | 597 | 6.8 | 4 | 10 | 68 |
| MFG | R | 10 | 1565 | 5.6 | 34 | 58 | -16 |
| IFG | R | 47 | 102 | 5.6 | 54 | 22 | -12 |
| SFG, MFG | L | 9, 10 | 650 | 5.4 | -26 | 50 | 30 |
| Superior Parietal Lobule | R | 7 | 646 | 5.4 | 36 | -64 | 54 |
| IFG | L | 47 | 332 | 5.4 | -56 | 22 | -10 |
| **IC20** | | | | | | | |
| Precuneus/PCC,  Inferior Parietal Lobule | L/R | 7, 23, 30,  31, 40 | 36096 | >10.0 | 10 | -58 | 14 |
| Frontal Eye Field | R | 8 | 889 | 6.3 | 26 | 16 | 54 |
| ITG | R | 21 | 153 | 5.6 | 62 | 0 | -16 |
| Frontal Eye Field | L | 8 | 594 | 6.0 | -24 | 6 | 50 |
| **IC22** | | | | | | | |
| MFG, IFG | L | 9, 46 | 5689 | >10 | -46 | 38 | 32 |
| MFG, IFG | R | 9, 46 | 6349 | 7.4 | 38 | 46 | 38 |
| Superior Parietal Lobule,  Inferior Parietal Lobule | L/R | 7, 19, 40 | 8262 | 6.8 | -4 | -76 | 48 |
| Insula | R | 13 | 177 | 5.8 | 42 | 14 | 6 |
| Insula | L | 13 | 196 | 5.6 | -34 | 24 | 8 |

The MNI (Montreal Neurological Institute) coordinates show the coordinates of the peak voxels. Clusters with k < 50 are not listed.

Abbreviations: ACC: anterior cingulate gyrus; G: gyrus; IFG: inferior frontal gyrus; L: left; MFG: middle frontal gyrus; mSFG: medial superior frontal gyrus; MTG: middle temporal gyrus; PCC: posterior cingulate gyrus; R: right; SFG: superior frontal gyrus.
