# Supplementary material for: Opposite Modulation of Brain Functional Networks Implicated at Low vs. High Demand of Attention and Working Memory
Source: PLoS One. 2014 Jan 31;9(1):e87078. doi: 10.1371/journal.pone.0087078 (PMC3909055; doi:10.1371/journal.pone.0087078)
Supplement: Table S2 — Beta weights of all ICs at each task condition and related p values. (DOC) [file pone.0087078.s004.doc]

Table S2 Beta weights of all ICs at each task condition and related p values

| **IC*** | 1 | 4 | 5 | 6 | 7 | 8 | 10 | 12 | 13 | 14 | 15 | 16 | 17 | 20 | 22 |
| --- | --- | --- | --- | --- | --- | --- | --- | --- | --- | --- | --- | --- | --- | --- | --- |
| **Mean beta weight values** | | | | | | | | | | | | | | | |
| **L** | -1.77  (1.11) | .25  (.47) | -.12  (.29) | .42  (1.2) | .11  (.21) | .13  (.56) | -1.05  (.75) | -.16  (.21) | -.77  (.71) | -.10  (.75) | -.19  (.38) | .75  (.73) | -.33  (.30) | -.42  (.40) | -.54  (.71) |
| **LD** | 1.21  (1.02) | .51  (.80) | .06  (.25) | .62  (1.2) | .12  (.24) | .03  (.24) | -.01  (.45) | -.17  (.25) | -.62  (.63) | -.10  (.42) | -.13  (.49) | .90  (.95) | -.19  (.26) | -.14  (.35) | -.50  (.58) |
| **H** | -1.61  (1.10) | -.77  (.78) | -.19  (.39) | -.90  (1.1) | -.30  (.22) | -.35  (.50) | -.45  (.37) | .10  (.15) | .78  (.61) | .11  (.31) | -.07  (.45) | -1.06  (.88) | .13  (.24) | -.51  (.59) | .31  (.35) |
| **HD** | 1.31  (.84) | -.80  (.74) | -.28  (.50) | -.80  (1.2) | -.37  (.26) | -.44  (.58) | .05  (.45) | .09  (.24) | .71  (.73) | -.01  (.40) | -.24  (.48) | -1.24  (1.08) | .21  (.26) | -.56  (.57) | .37  (.49) |
| **P values of one-sample t-test against zero** | | | | | | | | | | | | | | | |
| **L** | .000 | .018 | .060 | .112 | .020 | .265 | .000 | .002 | .000 | .110 | .030 | .000 | .000 | .000 | .002 |
| **LD** | .000 | .006 | .230 | .023 | .029 | .581 | .884 | .003 | .000 | .260 | .220 | .000 | .002 | .074 | .000 |
| **H** | .000 | .000 | .029 | .001 | .000 | .003 | .000 | .004 | .000 | .090 | .480 | .000 | .018 | .000 | .000 |
| **HD** | .000 | .000 | .015 | .006 | .000 | .001 | .592 | .092 | .000 | .920 | .026 | .000 | .001 | .000 | .002 |
| **P values of main effects of task load, distractors, and their interaction.** | | | | | | | | | | | | | | | |
| **LOA** | .12 | .000 | .14 | .005 | .000 | .010 | .009 | .001 | .000 | .24 | .97 | .000 | .000 | .042 | .000 |
| **DIST** | .000 | .21 | .28 | .32 | .46 | .15 | .000 | .61 | .53 | .25 | .56 | .80 | .053 | .20 | .47 |
| **INTER** | .66 | .02 | .01 | .73 | .43 | .94 | .01 | .95 | .04 | .21 | .14 | .18 | .49 | .08 | .90 |

*The first row shows the IC numbers. Numbers in parentheses are standard deviations (SD). Abbreviation: H: high load without distractors; HD: high load with distractors; L: low load without distractors; LD: low load with distractors; LOA: main effects of task load; DISA: main effects of distractors; INTER: effects of load and distractor interaction.
